# Predicting 90-day postoperative outcomes in spontaneous supratentorial intracerebral hemorrhage: a double-center study of clinical-radiomics integration using non-contrast CT

**DOI:** 10.3389/fneur.2025.1674276

**Published:** 2025-12-16

**Authors:** Wenli Jiang, Changgu Zhou, Jian Wang, Hong Xu, Zhiming Zhou

**Affiliations:** 1 Department of Radiology, The Second Affiliated Hospital of Chongqing Medical University, Chongqing, China; 2 People’s Hospital of Linshui County, Guang’an, China; 3 The Second Clinical College of Chongqing Medical University, Chongqing, China; 4 Chongqing Medical Imaging Artificial Intelligence Lab, Chongqing, China

**Keywords:** spontaneous intracerebral hemorrhage, radiomics, non-contrast computed tomography, prognosis, postoperative

## Abstract

**Objective:**

To evaluate the clinical utility of integrating baseline non-contrast computed tomography (NCCT) radiomics with clinical and surgical parameters for predicting 90-day postoperative functional outcomes in patients with spontaneous supratentorial intracerebral hemorrhage (sICH).

**Materials and methods:**

We retrospectively analyzed data from 220 patients with sICH who underwent surgical treatment from January 2022 to January 2025. Patients from Center 1 (*n* = 154) formed the training cohort, and those from Center 2 (*n* = 66) served as the validation cohort. Functional outcomes at 90 days were stratified using the modified Rankin Scale. Independent clinical risk factors were identified via univariate and multivariate analyses to construct a clinical model. Radiomics features extracted from baseline NCCT images were screened using elastic net regression with 10-fold cross-validation to generate a radiomics score (Radscore). A combined model was constructed by integrating Radscore into the clinical model, and its performance was evaluated using receiver operating characteristic curves, with visualization as a nomogram.

**Results:**

Six independent clinical risk factors (age, hydrocephalus, brain herniation, hematoma location, hematoma volume, surgical duration) and three optimal radiomics features (Flatness, Least axis length, VolumeCC) were identified. The combined model achieved the highest area under the receiver operating characteristic curve values: 0.882 in the training cohort and 0.865 in the validation cohort, outperforming the clinical model (0.844, 0.830) and Radscore (0.769, 0.743).

**Conclusion:**

NCCT radiomics integrated with clinical parameters effectively predicts 90-day postoperative outcomes in sICH. The combined model, visualized as a nomogram, aids preoperative risk stratification and personalized treatment.

## Introduction

Spontaneous intracerebral hemorrhage (ICH) is a neurological emergency with high morbidity and mortality, imposing a heavy burden on patients, families, and society (1). Early surgical interventions, including craniotomy evacuation of hematoma and minimally invasive puncture drainage, have emerged as important therapeutic strategies to improve the quality of life of ICH patients. These interventions achieve benefits by rapidly reducing intracranial pressure, alleviating hematoma mass effect, and mitigating secondary brain injury (2). However, clinically significant interindividual variability in postoperative functional recovery is commonly observed, which may stem from baseline clinical conditions, surgical variables, and hematoma characteristics (3–5). This variability underscores the critical need for individualized prognostic evaluations to inform clinical decision-making (1).

Baseline clinical features play a pivotal role in influencing surgical decision-making and treatment outcomes. Studies have identified age and neurological deficits as independent risk factors for poor postoperative outcomes in ICH patients (6). Additionally, surgical approaches are closely related to outcomes: craniotomy is more effective for evacuating superficial lobar hematomas, while minimally invasive techniques (e.g., stereotactic puncture) offer advantages in minimizing damage to healthy brain tissue (1, 7). Nevertheless, existing research has predominantly focused on comparing the efficacy of individual surgical techniques, with a paucity of systematic investigations into the association between procedural details (e.g., surgical duration, intraoperative blood loss, transfusion volume) and clinical outcomes.

Hematoma characteristics also exert a significant impact on functional outcomes after ICH. Traditional computed tomography (CT) parameters, such as hematoma volume, location, and intraventricular extension, have been incorporated into various prognostic scoring systems, including the Intracerebral Hemorrhage Scale and Intracerebral Hemorrhage Functional Outcome Scale (8). Hematoma heterogeneity, encompassing features like irregular morphology and density variations, has also been reliably shown to predict poor outcomes in ICH patients (9). In recent years, radiomics has facilitated the quantitative characterization of hematoma heterogeneity by extracting high-throughput texture, morphological, and histogram features from CT images, demonstrating promising efficacy in predicting 90-day poor postoperative outcomes in spontaneous ICH patients (10, 11). However, radiomics studies on postoperative functional outcomes have been restricted to specific surgical techniques (e.g., craniotomy or stereotactic hematoma removal), highlighting an unmet need for more comprehensive predictive tools.

To address these gaps, the present study systematically integrates patients’ baseline clinical data, key surgical parameters, and radiomics features extracted from baseline hematoma images to develop and validate a predictive model for 90-day poor postoperative outcomes in spontaneous supratentorial intracerebral hemorrhage (sICH) patients undergoing diverse surgical approaches. This model aims to provide robust support for clinicians in preoperative risk stratification, postoperative recovery prediction, and the optimization of personalized treatment strategies.

## Materials and methods

### Study patients and clinical data

Patients with spontaneous sICH who had completed surgical treatment were retrospectively enrolled from two centers: Center 1 (the Second Affiliated Hospital of Chongqing Medical University, Chongqing, China) and Center 2 (People’s Hospital of Linshui County, Guang’an, China), with enrollment spanning from January 2022 to January 2025. SICH was diagnosed based on non-contrast computed tomography (NCCT) findings demonstrating parenchymal hemorrhage. Inclusion criteria were: (1) sICH confirmed by preoperative NCCT; (2) patients who underwent surgical intervention within 72 h of symptom onset. Exclusion criteria included: (1) secondary hemorrhage due to trauma, aneurysms, arteriovenous malformations, cerebral infarction, intracranial tumors, or coagulation disorders; (2) isolated intraventricular hemorrhage or concurrent cerebellar/brainstem hemorrhage; (3) severe imaging artifacts (e.g., metal or motion artifacts) affecting analysis; (4) loss to follow-up or missing key clinical or imaging data. Center 1 served as the training cohort, and Center 2 as the validation cohort.

Baseline NCCT images and clinical data were collected retrospectively, including demographic characteristics, medical history, admission vital signs, and laboratory results. Postoperative modified Rankin Scale (mRS) scores at 90 days were obtained via telephone interviews follow-up or review of electronic medical records. Patients were categorized into two groups: favorable outcome (mRS 0–3), which included mRS 0 (no neurological deficits), 1 (minor stroke-related symptoms), 2 (mild disability), and 3 (moderate disability); and poor outcome (mRS 4–6), which included mRS 4 (severe disability), 5 (profound disability), and 6 (death).

### Surgical data

All patients were managed in accordance with the latest Chinese Guidelines for the Diagnosis and Treatment of Intracerebral Hemorrhage 2019 (12). For patients with lobar hemorrhage > 30 mL and located within 1 cm of the cortical surface, standard craniotomy for supratentorial hematoma evacuation or minimally invasive hematoma evacuation could be considered. When patients exhibited progressive neurological deterioration or life-threatening cerebral herniation, the attending chief or associate chief physician evaluated the necessity of surgery and determined the surgical approach. Included surgical modalities consisted of traditional craniotomy, microscopic hematoma evacuation, neuroendoscopic hematoma evacuation, and hematoma puncture or external ventricular drainage. Surgical data were collected for subsequent analysis, including time interval from preoperative NCCT to surgery, surgical timing (defined as the interval from symptom onset to surgery), surgical duration (defined as the total duration of the surgical procedure), intraoperative blood loss, and intraoperative blood transfusion volume.

### Imaging acquisition

NCCT scans were performed using CT scanners (Philips Medical Systems; Canon Medical Systems) with standardized acquisition parameters: tube voltage 120 kV, tube current 250–300 mA, matrix 512 × 512, gantry rotation time 0.4–0.6 s, field of view 25 cm, and slice thickness 1 mm. All images were digitally stored in the picture archiving and communication system (PACS) for subsequent analysis.

### Radiomics analysis

Image Segmentation: Baseline NCCT images were retrieved from PACS and exported in the format of digital imaging and communications in medicine. Hematoma regions were semi-automatically segmented using ITK-SNAP software (version 3.8.0, http://www.itksnap.org). Regions of interest (ROIs) were delineated by an experienced radiologist with ≥ 5 years of neuroimaging experience, who was blinded to clinical outcomes and follow-up data to avoid bias.

Image Preprocessing: To mitigate potential biases introduced by variations in scanning devices and acquisition parameters, NCCT images underwent preprocessing steps: (1) voxel resampling to a uniform spatial resolution of 1 × 1 × 1 mm^3^; (2) grayscale intensity discretization into 256 bins; (3) standardization of window settings to a width of 90 Hounsfield units (HU) and a level of 40 HU. These steps ensured consistent scaling and spatial resolution across all images, enhancing the comparability of extracted radiomics features.

### Feature extraction

Radiomics feature extraction was performed using Python software (version 3.2). A total of 107 radiomics features were automatically extracted from each ROI, encompassing eight categories: 2D morphological features, 3D morphological features, first-order statistics, gray-level co-occurrence matrix, gray-level run-length matrix, gray-level size zone matrix, gray-level dependence matrix, and neighborhood gray-tone difference matrix.

To assess the stability of segmentation and feature extraction, 30 randomly selected NCCT images were re-annotated and re-analyzed by the same radiologist after 4 weeks interval and by another radiologist (with 2 years of working experience) using the same segmentation protocol using ITK-SNAP. Radiomics features were extracted from all sets of segmentations (the original, the intra-observer repeat, and the two inter-observer). Intraclass correlation coefficients (ICCs) were computed to evaluate feature consistency, with features exhibiting ICC ≥ 0.75 considered stable. Optimal features were further selected via elastic net regression with 10-fold cross-validation to reduce dimensionality and avoid overfitting.

### Model construction and validation

In the training cohort, univariate analyses were first performed to identify clinical factors associated with 90-day poor outcomes, with factors showing *p* < 0.1 included in subsequent multivariate analysis. Features with statistical significance in multivariate analysis (*p* < 0.05) were retained to construct a clinical prediction model using logistic regression.

The radiomics score (Radscore) was calculated using the formula: Radscore $=(\sum_{i=1}\beta_{i}*F_{i})$ , where $F_{i}$ denotes the i-th radiomics feature and $\beta_{i}$ represents its corresponding coefficient derived from elastic net regression. A combined model was then developed using logistic regression, by integrating the Radscore with the significant independent clinical risk factors identified from the multivariate analysis.

The combined model, Radscore and clinical model were validated in the validation cohort.

### Statistical analysis

Statistical analyses were performed using SPSS software (version 19.0; IBM Corp., Armonk, NY, United States). Continuous data with normal distribution were presented as mean ± standard deviation and compared using independent samples t-tests; non-normally distributed continuous data were expressed as median (interquartile range) and compared using the Mann–Whitney U test. Categorical data were presented as counts (percentages) and compared using the χ^2^ test or Fisher’s exact test when expected cell counts were < 5.

Receiver operating characteristic (ROC) curves and AUCs were used to evaluate the predictive performance of the clinical model, Radscore, and the combined model. Differences in AUC between models were compared using the DeLong test. The optimal model was visualized as a nomogram. Calibration curves and decision curve analysis (DCA) were used to evaluate the model’s calibration and clinical utility, respectively. Statistical significance was set at *p* < 0.05.

## Results

### Clinical characterization

A total of 457 patients were screened at Center 1 (training cohort) and 224 at Center 2 (validation cohort), with 154 and 66 patients ultimately included, respectively. Exclusion criteria were consistent across both centers, with excluded patients distributed by reason as follows: secondary hemorrhage (212 in Center 1 vs. 104 in Center 2), isolated intraventricular or concurrent cerebellar/brainstem hemorrhage (40 vs. 18), severe imaging artifacts (6 vs. 4), and incomplete baseline data (45 vs. 32). In total, 303 patients were excluded from Center 1 and 158 from Center 2.

The distribution of surgical approaches in the training and validation cohorts was as follows: traditional craniotomy (33 [21.4%] vs. 11 [16.7%]), microscopic hematoma evacuation (48 [31.2%] vs. 20 [30.3%]), neuroendoscopic evacuation (32 [20.8%] vs. 11 [16.7%]), and hematoma puncture or external ventricular drainage (41 [26.6%] vs. 24 [36.4%]). No significant differences were observed in clinical data between the training and validation cohorts (all *p* > 0.05), indicating good consistency in patient demographics across cohorts (Table 1).

**Table 1:** Baseline data of sICH patients in the center 1 and center 2.

Variables	Center 1 (*n* = 154)	Center 2 (*n* = 66)	*P*
Age (y)	58.85 ± 14.28	59.23 ± 13.34	0.855
Male/Female	115/39	49/17	1.000
Hypertension	93 (60.4%)	47 (71.2%)	0.169
Coronary heart disease	10 (6.5%)	7 (10.6%)	0.440
Diabetes	16 (10.4%)	9 (13.6%)	0.643
Temperature (°C)	36.5 (36.50, 36.68)	36.5 (36.50, 36.70)	0.640
Systolic blood pressure (mmHg)	172.80 ± 30.88	172.12 ± 29.36	0.880
Diastolic blood pressure (mmHg)	98.88 ± 19.32	101.584 ± 18.86	0.340
GCS score	9.00 (6.25, 13.00)	8.00 (6.00, 12.00)	0.447
Pupil abnormality	70 (45.5%)	33 (50.0%)	0.637
Blood glucose (mmol/L)	7.94 (6.80, 9.66)	8.38 (6.93, 9.40)	0.651
D-Dimer (mg/L)	345.85 (150.48, 799.40)	420.00 (188.73, 951.42)	0.477
C-reactive protein (mg/L)	3.31 (1.29, 6.50)	3.45 (1.99, 10.52)	0.105
Neutrophil-lymphocyte ratio	10.30 (6.44, 16.95)	12.37 (7.35, 19.83)	0.233
Hemoglobin (g/L)	133.51 ± 21.13	135.03 ± 20.66	0.642
Platelet count (10^9^/L)	197.60 ± 65.23	201.55 ± 54.56	0.667
Activated partial Thromboplastin time (s)	33.20 (30.72, 35.77)	33.05 (31.42, 35.50)	0.858
International normalized ratio	0.98 (0.93, 1.03)	0.98 (0.93, 1.03)	0.889
Fibrinogen (mg/dL)	2.98 ± 0.76	3.10 ± 0.67	0.280
Hematoma volume	50.90 (39.08, 76.92)	46.45 (28.47, 70.50)	0.222
Hematoma location			0.918
Lobar hematoma	37 (24.0%)	17 (25.8%)	
Deep hematoma	117 (76.0%)	49 (74.2%)	
Hydrocephalus	39 (25.3%)	20 (30.0%)	0.550
Subarachnoid hemorrhage	26 (16.9%)	9 (13.6%)	0.688
Intraventricular hemorrhage	92 (59.7%)	35 (53.0%)	0.439
Brain herniation	36 (23.4%)	20 (30.3%)	0.362
Midline shift	70 (45.5%)	30 (45.5%)	1.000
Time interval from NCCT to surgery (h)	4.00 (2.00, 7.00)	4.00 (3.00, 8.00)	0.654
Surgical approach			0.467
Minimally invasive surgery	121 (78.6%)	55 (83.3%)	
Craniotomy	33 (21.4%)	11 (16.7%)	
Surgical duration (h)	3.09 ± 1.77	3.08 ± 1.76	0.984
Intraoperative bleeding (mL)	122.00 (52.00, 422.00)	200.00 (36.75, 422.00)	0.939
Blood transfusion (mL)	56.86 ± 159.68	36.82 ± 113.25	0.356
mRS score	4.00 (3.00, 4.00)	4.00 (3.00, 4.75)	0.553
Poor outcome	95 (61.7%)	43 (65.2%)	0.738
Early rehabilitation	32 (20.8%)	8 (12.1%)	0.182
Flatness	0.23 ± 0.99	0.21 ± 1.00	0.898
Least axis length	0.05 ± 0.90	0.26 ± 1.09	0.127
VolumeCC	0.06 ± 0.05	0.05 ± 0.03	0.251
Radscore	20.79 ± 77.11	20.24 ± 94.54	0.964

Data are expressed as mean ± standard deviation, median (1st quartile, 3rd quartile), or frequency (percentage).

In the training cohort (*n* = 154), 95 patients (62.3%) experienced poor 90-day outcomes. Detailed baseline characteristics are summarized in Table 2. Compared with the favorable outcome subgroup in the training cohort, patients with poor outcomes exhibited several distinct features: older age (*p* = 0.006); lower admission Glasgow Coma Scale (GCS) scores (*p* < 0.001); reduced preoperative hemoglobin levels (*p* = 0.001); larger hematoma volumes (*p* < 0.001); a higher proportion of deep-seated hematomas (*p* = 0.004); shorter time intervals from NCCT to surgery (*p* = 0.029); longer surgical duration (*p* = 0.017); greater intraoperative blood loss (*p* = 0.009); higher mRS scores (*p* < 0.001); and increased rates of pupillary abnormalities (*p* = 0.002), hydrocephalus (*p* = 0.001), brain herniation (*p* < 0.001), and midline shift (*p* = 0.015).

**Table 2:** Baseline data of sICH patients in the training cohort.

Variables	Poor outcome (*n* = 95)	Favorable outcome (*n* = 59)	*P*
Age (y)	61.35 ± 13.64	54.83 ± 14.49	0.006
Male/Female	68/27	47/12	0.352
Hypertension	63 (66.3%)	30 (50.8%)	0.082
Coronary heart disease	8 (8.4%)	2 (3.4%)	0.371
Diabetes	12 (12.6%)	4 (6.8%)	0.376
Temperature (°C)	36.5 (36.5, 36.7)	36.5 (36.5, 36.6)	0.070
Systolic blood pressure (mmHg)	174.33 ± 32.64	170.34 ± 27.91	0.438
Diastolic blood pressure (mmHg)	97.97 ± 19.15	100.34 ± 19.66	0.461
GCS score	8.00 (6.00, 11.00)	12.00 (9.00, 13.5)	<0.001
Pupil abnormality	53 (55.8%)	17 (28.8%)	0.002
Blood glucose (mmol/L)	8.45 (7.00, 9.86)	7.67 (6.35, 9.07)	0.054
D-Dimer (mg/L)	361.30 (160.55, 1163.25)	292.30 (142.70, 579.50)	0.173
C-reactive protein (mg/L)	3.32 (1.44, 6.33)	3.30 (1.05, 7.14)	0.676
Neutrophil-lymphocyte ratio	10.10 (6.84, 16.90)	10.52 (5.94, 16.71)	0.595
Hemoglobin (g/L)	129.18 ± 22.27	140.49 ± 17.12	0.001
Platelet count (10^9^/L)	194.25 ± 70.73	202.98 ± 55.40	0.421
Activated partial thromboplastin time (s)	33.30 (30.90, 36.00)	33.20 (30.70, 35.10)	0.433
International normalized ratio	0.99 (0.93, 1.05)	0.97 (0.93, 1.00)	0.220
Fibrinogen (mg/dL)	3.02 ± 0.87	2.91 ± 0.55	0.388
Hematoma volume	57.50 (43.30, 87.40)	44.70 (27.90, 57.00)	<0.001
Hematoma location			0.004
Lobar hematoma	15 (15.8%)	22 (37.3%)	
Deep hematoma	80 (84.2%)	37 (62.7%)	
Hydrocephalus	33 (34.7%)	6 (10.2%)	0.001
Subarachnoid hemorrhage	16 (16.8%)	10 (16.9%)	1.000
Intraventricular hemorrhage	63 (66.3%)	29 (49.2%)	0.052
Brain herniation	33 (34.7%)	3 (5.1%)	<0.001
Midline shift	51 (53.7%)	19 (32.2%)	0.015
Time interval from NCCT to surgery (h)	4.00 (2.00, 6.00)	5.00 (3.00, 9.00)	0.029
Surgical approach			0.319
Minimally invasive surgery	72 (75.8%)	49 (83.1%)	
Craniotomy	23 (24.2%)	10 (16.9%)	
Surgical duration (h)	3.36 ± 1.87	2.66 ± 1.53	0.017
Intraoperative bleeding (mL)	222.00 (103.50, 422.00)	122.00 (22.00, 322.00)	0.009
Blood transfusion (mL)	67.96 ± 172.91	38.98 ± 135.21	0.275
mRS score	4.00 (4.00, 5.00)	3.00 (2.00, 3.00)	<0.001
Early rehabilitation	21 (22.1%)	11 (18.6%)	0.756
Flatness	0.52 ± 0.92	−0.24 ± 0.93	<0.001
Least axis length	0.24 ± 0.83	−0.26 ± 0.92	0.001
VolumeCC	0.07 ± 0.05	0.04 ± 0.02	0.001
Radscore	48.33 ± 64.63	−23.55 ± 75.33	<0.001

Data are expressed as mean ± standard deviation, median (1st quartile, 3rd quartile), or frequency (percentage).

Multivariate analysis identified six independent predictors of poor 90-day outcomes: presence of hydrocephalus (*p* = 0.023), presence of brain herniation (*p* = 0.040), hematoma location (*p* = 0.027), hematoma volume (*p* = 0.016), and surgical duration (*p* = 0.025) (Table 3).

**Table 3:** Multivariate analysis for poor outcome in the training cohort.

Variables	OR	95% CI	*P*
Age	1.040	1.005 ~ 1.077	0.026
GCS score	0.903	0.768 ~ 1.062	0.219
Pupil abnormality	1.783	0.623 ~ 5.104	0.281
Hemoglobin	0.980	0.958 ~ 1.003	0.087
Hydrocephalus	0.236	0.068 ~ 0.823	0.023
Brain herniation	0.179	0.035 ~ 0.923	0.040
Midline shift	0.917	0.336 ~ 2.500	0.865
Hematoma location	0.309	0.109 ~ 0.875	0.027
Hematoma volume	1.030	1.006 ~ 1.055	0.016
Time interval from NCCT to surgery	0.989	0.944 ~ 1.036	0.630
Surgical duration	1.549	1.057 ~ 2.271	0.025
Intraoperative bleeding	0.999	0.996 ~ 1.002	0.385

### Radiomics analysis

Elastic net regression with 10-fold cross-validation was used to select optimal radiomics features from the training cohort, resulting in three stable features: Flatness, Least axis length, and VolumeCC. These features were incorporated to calculate the Radscore.

In the training cohort, the mean Radscore was significantly lower in the favorable outcome subgroup (−23.55 ± 75.33) compared with the poor outcome subgroup (48.33 ± 64.63; *p* < 0.001). A consistent pattern was observed in the validation cohort: the favorable outcome subgroup had a lower mean Radscore (−30.39 ± 77.31) than the poor outcome subgroup (47.33 ± 92.44; *p* = 0.001). After adjusting for clinical risk factors, Radscore remained an independent predictor of poor 90-day outcomes in sICH patients (odds ratio [OR] = 1.015; 95% confidence interval [CI]: 1.007–1.023; *p* < 0.001).

Reproducibility analyses confirmed the stability of both segmentations and radiomics features. Intra- and inter-observer analyses demonstrated that 104/107 (97.2%) and 100/107 (93.5%) features, respectively, exhibited ICCs greater than 0.75. All three features constituting the Radscore showed good reproducibility: Flatness (intra-observer ICC = 0.97 [95% CI: 0.942–0.987]; inter-observer ICC = 0.96 [95% CI: 0.911–0.979]), Least axis length (intra-observer ICC = 0.95 [95% CI: 0.889–0.974]; inter-observer ICC = 0.92 [95% CI: 0.840–0.961]), and VolumeCC (intra-observer ICC = 0.94 [95% CI: 0.870–0.969]; inter-observer ICC = 0.91 [95% CI: 0.828–0.958]). Critically, the composite Radscore itself also demonstrated exceptional intra-observer stability and inter-observer stability, with ICCs of 0.97 [95% CI: 0.930–0.984] and 0.95 [95% CI: 0.894–0.975].

### Model prediction performance

**Figure 1:**
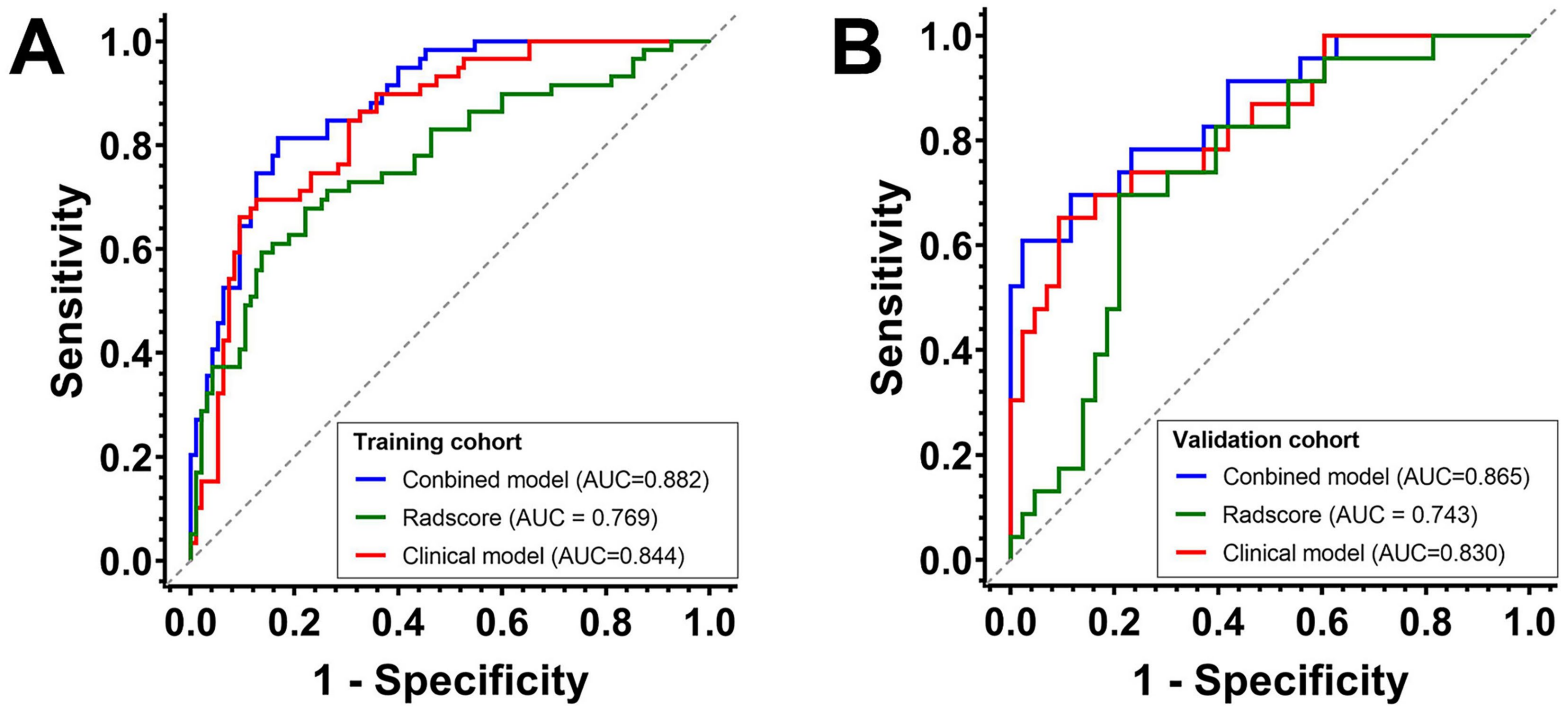
ROC curves of the predictive model in the training cohort **(A)** and the validation cohort **(B)**.

The combined model (integrating clinical factors and Radscore) showed the highest predictive performance (Figure 1), with AUCs of 0.882 (95% CI: 0.820–0.928) in the training cohort and 0.865 (95% CI: 0.758–0.936) in the validation cohort. The clinical model alone achieved AUCs of 0.844 (95% CI: 0.777–0.898) in the training cohort and 0.830 (95% CI: 0.718–0.911) in the validation cohort, while Radscore alone yielded AUCs of 0.769 (95% CI: 0.694–0.833) and 0.743 (95% CI: 0.621–0.843) in the training and the validation cohorts, respectively.

DeLong tests revealed that the combined model had a significantly higher AUC than Radscore alone in both the training cohort (*p* = 0.002) and the validation cohort (*p* = 0.044). However, no significant differences in AUC were observed between the combined model and the clinical model in either the training cohort (*p* = 0.068) or the validation cohort (*p* = 0.262). Similarly, no significant differences were found between the clinical model and Radscore in the training cohort (*p* = 0.140) or the validation cohort (*p* = 0.268).

### Nomogram and evaluation curves

**Figure 2:**
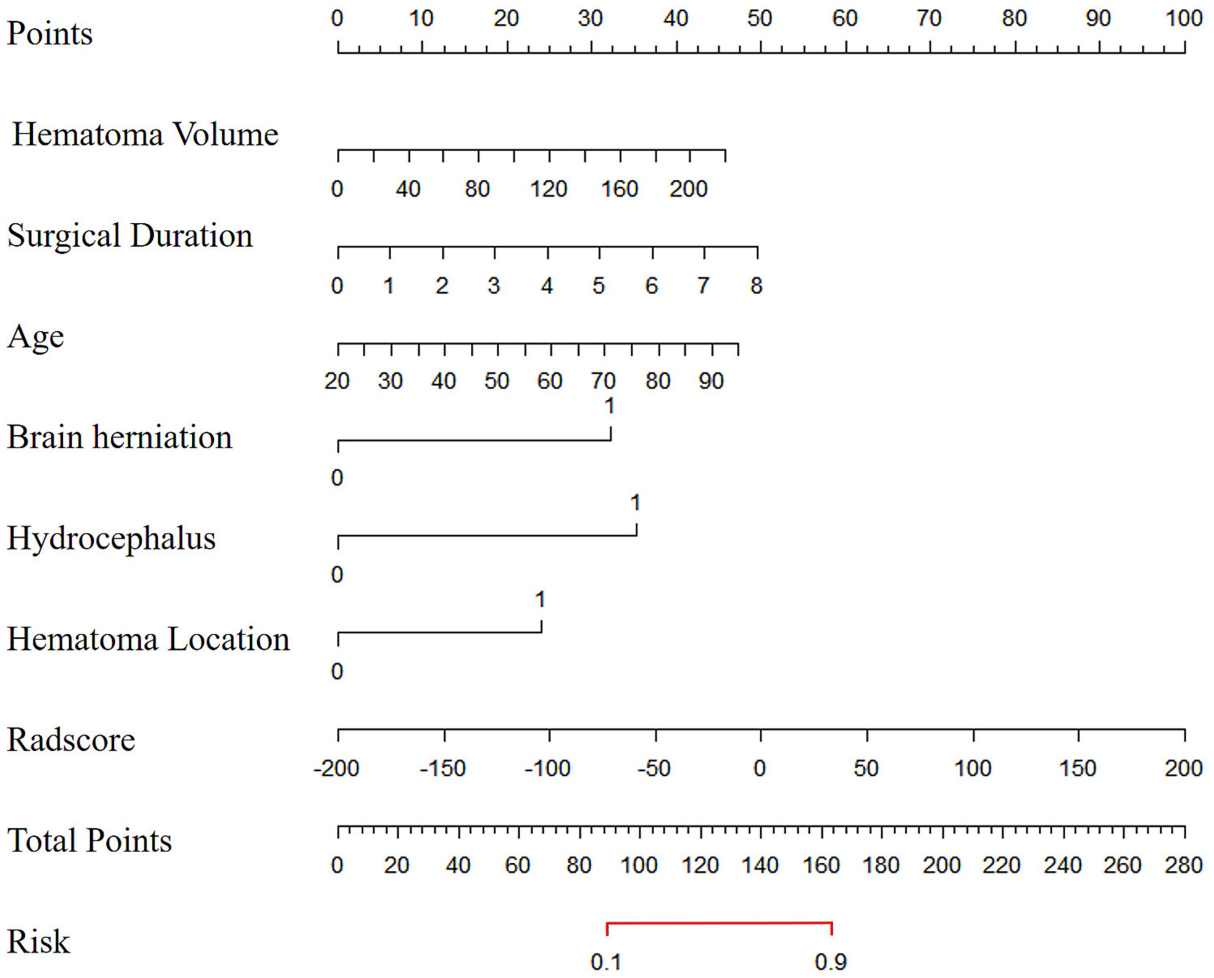
The nomogram diagram of the combined model.

**Figure 3:**
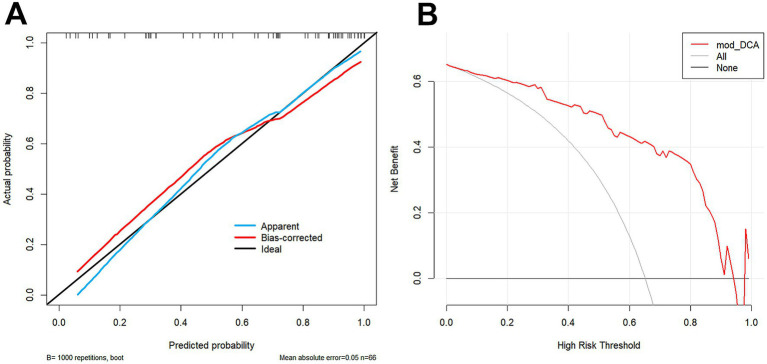
Calibration curves **(A)** and decision curves **(B)** of the combined model in the validation cohort.

The combined model was visualized as a user-friendly nomogram to facilitate clinical application (Figure 2). Calibration curves demonstrated good agreement between predicted probabilities of poor 90-day outcomes and observed outcomes in both the training and the validation cohorts (Figure 3A). DCA confirmed that the combined model provided a high net benefit across a range of threshold probabilities, supporting its clinical utility (Figure 3B).

## Discussion

This double-center study advances the field of sICH prognosis by systematically examining the relationships between baseline clinical characteristics, surgical parameters, hematoma radiomics features, and 90-day postoperative functional outcomes. We identified six independent clinical risk factors and three optimal radiomics features, then developed multiple predictive models for 90-day outcomes. Notably, the combined model demonstrated superior predictive performance, offering a practical tool to identify sICH patients at high risk of poor postoperative outcomes and support clinical decision-making.

Our analysis revealed that age, hydrocephalus, brain herniation, hematoma location, hematoma volume, and surgical duration are independent risk factors for poor 90-day postoperative outcomes in sICH patients. A key novel finding is that surgical duration emerges as an independent predictor of adverse functional outcomes, a relationship not previously highlighted in sICH research. Prolonged surgery may heighten the risks of surgical site infection (13) while exacerbating mechanical trauma or ischemia–reperfusion injury to brain tissue. This observation underscores the importance of preoperative planning: clinicians should estimate expected durations for different surgical approaches (e.g., craniotomy vs. minimally invasive puncture) using clinical and imaging data, then select strategies to minimize surgical time and tailor individualized plans, potentially improving postoperative recovery.

Consistent with prior studies (3), age was confirmed as an independent risk factor, likely due to age-related declines in organ reserve, higher burdens of comorbidities such as hypertension and hyperglycemia, and reduced surgical tolerance and recovery capacity in older patients. Hydrocephalus, a common sICH complication, was also linked to poor outcomes, aligning with findings by Bhattathiri et al. (14); its pathogenic mechanism may involve secondary injury from disrupted cerebrospinal fluid circulation. Brain herniation, by compressing cranial nerves and vasculature, can induce hemorrhage or ischemia, worsening neurological damage, and impairing outcomes (15).

We further observed that deep-seated hematomas correlate with worse functional outcomes compared to superficial hematomas, consistent with earlier reports (16). This may reflect greater vulnerability of functional fiber bundles to deep hemorrhage. For example, thalamic sICH often involves intraventricular extension and brainstem compression, exacerbating motor dysfunction (17). Larger baseline hematoma volumes also predicted poor outcomes, supporting previous evidence that hematoma size correlates with neurological injury severity (18). Collectively, these findings highlight that preoperative assessment of clinical and imaging features, including estimated surgical duration, can inform surgical planning to optimize patient outcomes.

### Discussion on radiomics analysis results

From 107 radiomics features extracted from baseline NCCT images, we identified three optimal features, including Flatness, Least axis length, and VolumeCC, that strongly correlate with 90-day poor outcomes. The Flatness, which quantifies the ratio of the largest to smallest principal components of hematoma shape, reflects the degree of hematoma flattening in a given plane; higher values indicate more pronounced flattening. This feature has previously been shown to outperform some clinical factors in predicting postoperative outcomes (10), underscoring its value in capturing subtle morphological characteristics relevant to prognosis. The Least axis length, derived from the shortest axis of the ellipse enclosing the ROI, was validated as a prognostic marker, consistent with Zhang et al.’s work linking this feature to 180-day outcomes in spontaneous thalamic hemorrhage (19). The VolumeCC, calculated by multiplying ROI voxel count by single voxel volume, offers advantages over traditional methods like the ABC/2 formula; as noted by Alexander et al. (20), computer-assisted volume analysis better supports surgical decision-making by improving accuracy in evaluating indications and midline shift.

Notably, our feature selection yielded a parsimonious set of three features, a number fewer than the 5–8 predictors in many previous hemorrhage prognostic models (10), yet these features demonstrated strong statistical significance as independent risk factors. Together, they quantify hematoma morphology across multiple dimensions, with significant positive correlations with 90-day poor outcomes, aligning with Li et al.’s theory that hematoma morphological characteristics influence clinical prognosis (21). This quantitative framework provides clinicians with objective evidence to refine surgical indication assessment and develop personalized treatment plans.

### Discussion on the performance of the prediction model

Historically, sICH outcome prediction has relied primarily on clinical indicators. For example, a deep learning-based model for hypertensive ICH, incorporating 64 clinical variables, achieved an AUC of 0.997 in the training cohort and 0.884 in the validation cohort, outperforming traditional scoring systems (22). In recent years, radiomics has enhanced predictive accuracy; Radscore has been validated as an independent predictor of hematoma expansion (23), and Zhang et al. developed a prognostic model with an AUC of 0.870 that remained robust in multicenter validation (10).

Integrating radiomics features with clinical variables enhances model performance, a finding supported by Huang et al., who demonstrated that adding radiomics features significantly improved the predictive accuracy of clinical models (24). In the current study, we constructed a Radscore using three selected features (Flatness, Least axis length, and VolumeCC) and then developed a combined model by integrating this Radscore with key clinical predictors. This combined model achieved AUC values of 0.882 in the training cohort and 0.865 in the validation cohort, outperforming the standalone Radscore model (0.769, 0.743) and the clinical model (0.844, 0.830). These results align with Zhang et al.’s observations that models integrating radiomics scores with clinical factors outperform single-modality models (19), highlighting the value of combining clinical insights with intrinsic hematoma features. Viet et al. similarly emphasized the superiority of clinical-imaging combined models, noting the limitations of relying solely on radiomics features (25), a conclusion reinforced by our data confirming the combined model’s stronger discriminative ability. This integrative approach represents a more effective predictive strategy, supporting precision medicine and optimizing surgical decision-making. Radiomics captures subtle heterogeneity in hematoma morphology, providing rich imaging-derived information to inform surgical planning. Additionally, our combined model exhibits strong calibration and clinical applicability; its conversion into a nomogram facilitates preoperative individualized risk assessment, making it a practical tool for clinical use. Based on the model’s predictions, patients with a favorable prognosis may confidently undergo standard surgical evacuation, whereas high-risk patients could be considered for less invasive strategies to minimize trauma, with the acceptance of potentially less complete evacuation.

### Limitations

This study has several limitations. First, its retrospective design and relatively small sample size may restrict the model’s external validity. Although the statistical methods employed are commonly used, future studies could benefit from more advanced variable selection techniques to improve model stability. Second, several potentially influential variables were not included, such as anesthesia modality, intraoperative blood pressure management, congestive cardiac failure, cerebral amyloid angiopathy, prior use of anticoagulant or antiplatelet medications, and mean continuous blood pressure measurements from intensive care or stroke unit settings. Their absence may affect the predictive accuracy of the model. Future prospective studies should emphasize standardized collection of these comprehensive clinical variables across participating centers to improve the generalizability and clinical utility of predictive models. Third, the radiomics workflow remains a significant barrier to clinical translation. The reliance on manual feature extraction using Python-based tools renders the process time-consuming and poorly standardized. Future efforts should therefore focus on developing streamlined, automated solutions to facilitate practical implementation in routine clinical settings.

## Conclusion

The combined model constructed in this study can effectively predict 90-day postoperative outcomes in sICH patients. By integrating clinical risk factors with quantitative radiomics features, this model supports preoperative risk stratification and personalized treatment planning, offering valuable clinical utility for optimizing sICH management.

## Data Availability

The original contributions presented in the study are included in the article/supplementary material, further inquiries can be directed to the corresponding author.
